# Understanding Depression in Women Across the Lifespan: Hormonal Insights From Brain Imaging

**DOI:** 10.1002/brb3.71463

**Published:** 2026-05-24

**Authors:** Ying Liu, Zhirun Chen, Rong Zi, Chongyi Zhao

**Affiliations:** ^1^ Medical School Kunming University of Science and Technology Kunming Yunnan Province China; ^2^ Department of Gynecology The First People's Hospital of Yunnan Province, The Affiliated Hospital of Kunming University of Science and Technology Kunming Yunnan Province China

**Keywords:** lifespan, multimodal magnetic resonance imaging, womentimodal magne

## Abstract

Depression represents a prevalent psychiatric condition, with women exhibiting a substantially higher incidence compared to men. Across various life stages, including adolescence, the perinatal period, perimenopause, and older adulthood, fluctuations in hormonal, psychological, and social factors contribute to distinct pathogenic mechanisms and clinical presentations of depression in women. Multimodal magnetic resonance imaging (MRI) has emerged as a valuable modality for investigating the neural substrates associated with these stage‐specific differences. This review outlines the current applications and advancements in MRI‐based research aimed at understanding depression in women across the lifespan.

## Graphical abstract

This graphical abstract illustrates a lifespan model of women's depression across adolescence, perinatal, perimenopausal, and late‐life stages. It integrates three dimensions: hormonal fluctuations, multimodal MRI alterations (hippocampus, amygdala, prefrontal cortex), and stage‐specific pathophysiological pathways. The model highlights how reproductive endocrine transitions interact with distinct neural circuit dysfunctions to shape depression etiology. It provides a roadmap for understanding female‐specific risk mechanisms and developing targeted early screening and personalized interventions.

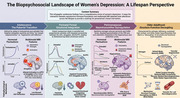

## 1 Introduction

Depression is a psychiatric disorder that exerts a substantial impact on both mental and physical health, with its global prevalence continuing to rise. (Richards 2011) It affects more than 264 million individuals worldwide and constitutes a major contributor to increased mortality risk. [(World Health Organization 2019, Xia et al. 2022)] The pathogenesis of depression involves multifaceted interactions among hormonal changes, genetic predispositions, and environmental exposures.

The prevalence of depression is significantly higher in women than in men, with estimates indicating nearly a twofold increase in risk among women. (Kuehner 2017, Kuehner 2003) This sex disparity is associated with several contributing factors, including fluctuations in sex hormones, sociocultural role expectations, and psychological stressors. The risk of depression is particularly heightened during periods of pronounced hormonal change, like adolescence, the postpartum period, and perimenopause. (Studd 2015) From a biological perspective, women of reproductive age exhibit notable differences in circulating levels of estrogen, progesterone, and testosterone when compared to men. These hormonal variations begin to emerge during adolescence, further accentuating the sex‐related divergence in depression prevalence. (Rubinow and Schmidt 2019)

Depression in women demonstrates variability in risk, clinical presentation, and potential underlying mechanisms across distinct life stages, including adolescence, the postpartum period, perimenopause, and older adulthood. However, the etiological factors contributing to these stage‐specific differences remain insufficiently characterized and warrant further investigation. In recent years, the use of multimodal magnetic resonance imaging (MRI) in depression research has expanded, providing a robust approach for exploring the neurobiological substrates of depression in women across the lifespan. This imaging modality encompasses structural MRI (sMRI), functional MRI (fMRI), diffusion tensor imaging (DTI), and magnetic resonance spectroscopy (MRS), facilitating a multidimensional assessment of brain alterations in individuals with depression ranging from anatomical structure and functional connectivity to metabolic activity. (Nugent et al. 2019)

This review highlights the current applications and recent advancements in MRI‐based research focused on depression in women across various life stages, with particular emphasis on imaging features and potential pathophysiological mechanisms during adolescence, the postpartum period, perimenopause, and older adulthood.

## 2 Classification of Multimodal MRI and its Applications in Depression

### 2.1 Functional MRI

The fMRI provides an indirect measure of neuronal activity by identifying fluctuations in cerebral blood oxygenation, a phenomenon known as the blood oxygen level‐dependent (BOLD) effect. This imaging modality facilitates the identification of brain activation patterns associated with specific tasks or psychological states (Logothetis 2008).

In the context of depression research, fMRI is frequently used to identify functional abnormalities in brain regions implicated in emotional regulation, reward processing, and cognitive control. Individuals with depression commonly exhibit aberrant activation in emotion‐related regions, including the amygdala, anterior cingulate cortex, and ventromedial prefrontal cortex, particularly in response to negative emotional stimuli. (Stickel et al. 2019) During reward‐related tasks, depression is associated with reduced activation in the ventral striatum, accompanied by increased activity in the medial prefrontal cortex and ventromedial frontal cortex (Zhang et al. 2013, Borsini et al. 2020).

Furthermore, fMRI serves as a useful tool for distinguishing bipolar depression (BD) from unipolar depression. These two conditions demonstrate distinct patterns of brain activation and functional connectivity during emotional processing, reward‐based paradigms, and working memory tasks. Notable differences have been observed in regions like the amygdala, anterior cingulate cortex, default mode network (DMN), and frontoparietal network. For example, during emotional processing tasks, patients with BD exhibit heightened prefrontal‐striatal activation during spatial planning. Additionally, during working memory tasks, BD is characterized by insufficient deactivation of the DMN (Han et al. 2019).

Despite its advantages, fMRI has certain limitations. The BOLD signal reflects the aggregated activity of large populations of neurons, which constrains their ability to capture neuronal activity at the level of individual cells.

### 2.2 Structural MRI

The sMRI primarily observes anatomical structure and volumetric changes of brain tissue. Through different imaging parameters and pulse sequences, high‐contrast images can be obtained to assess characteristics like gray and white matter volumes and cortical thickness. In depression research, sMRI identifies macroscopic brain structural changes, like hippocampal volume reduction and prefrontal cortex atrophy. However, because it cannot directly reflect neuronal activity or metabolic status, sMRI has limitations in presenting microscopic pathological changes (Lerch et al. 2017, Waller et al. 2021).

### 2.3 Diffusion MRI

Diffusion magnetic resonance imaging (dMRI) assesses microstructural tissue changes by measuring the Brownian motion of water molecules within biological tissues. The dMRI encompasses both diffusion‐weighted imaging (DWI) and DTI. DWI captures the overall diffusion of water molecules within tissues and is primarily employed for lesion identification, whereas DTI quantifies the anisotropic diffusion of water, facilitating the visualization and analysis of white matter fiber tracts (Basser and Pierpaoli 2011).

The dMRI is valuable in depression research and is frequently used to examine white matter integrity. It is particularly effective in identifying compromised fiber connectivity or reduced structural coherence in regions like the corpus callosum, internal capsule, prefrontal cortex, and parietal lobe (Hyett et al. 2018). In addition, dMRI techniques like diffusional kurtosis imaging have presented notable microstructural abnormalities in several brain regions among individuals with depression, including the right superior temporal gyrus, bilateral posterior thalamic radiation, and left middle cerebellar peduncle (Ota et al. 2018). These abnormalities are hypothesized to be associated with emotional dysregulation and psychomotor slowing observed in depressive disorders.

Currently, research specifically using dMRI to examine the lifespan‐related characteristics of depression in women remains limited. Most existing evidence is derived from broader studies that integrate dMRI with other neuroimaging methods.

### 2.4 Magnetic Resonance Spectroscopy

MRS is a non‐invasive technique used to measure concentrations of brain metabolites. It identifies and quantifies specific metabolites by assessing the resonance frequencies of hydrogen nuclei within varying chemical environments in a magnetic field (Faghihi et al. 2017). In individuals with depression, MRS commonly presents reduced levels of N‐acetylaspartate, choline, glutamate complexes, and γ‐aminobutyric acid in regions like the prefrontal cortex, hippocampus, and cingulate cortex, indicating potential impairments in energy metabolism and neurotransmitter synthesis (Benson et al. 2020). Additionally, elevated levels of glutamate and myo‐inositol have been observed in the basal ganglia of individuals with depression, findings that are associated with activation of the kynurenine pathway via inflammatory processes (Chen et al. 2021). MRS thus serves as a valuable tool for exploring the neurochemical mechanisms underlying depression.

## 3 Characteristics of Multimodal Magnetic Resonance Imaging in Depression Across the Female Lifespan

### 3.1 Adolescent Depression

Adolescence represents a key period of development marked by rapid physical and psychological transformations. Evidence indicates that prior to puberty, the prevalence of depression and anxiety disorders is slightly higher among males; however, after the age of 12, the incidence among females increases markedly and surpasses that of males (Wesselhoeft et al. 2015). During adolescence, emotional disorders primarily manifest as anxiety and depressive conditions, with severe cases potentially progressing to suicidal ideation. Adolescent females undergo psychological changes, including accelerated cognitive maturation and greater autonomy, which influence emotional regulation and elevate the risk of mood disorders (Shorey et al. 2022). When compared to adults, depressive episodes in adolescents are more frequently characterized by vegetative symptoms like changes in appetite, weight, and sleep patterns, whereas adults more commonly present with anhedonia and impaired concentration (Rice et al. 2019, Bernaras et al. 2019).

Significant fluctuations in sex hormone levels during puberty constitute a key biological factor contributing to the increased risk of depression among adolescent females. Evidence indicates a significant association between elevated levels of testosterone and estradiol and the risk of depression in this population. Specifically, testosterone concentrations exceeding 24.7 ng/dL have been linked to a significantly increased risk of depression, while estradiol, although exerting a relatively modest effect presents a positive correlation with depressive symptoms (Angold et al. 1999). Additional studies have demonstrated that elevated testosterone levels are associated with both depression and sleep disturbances, and that complex interactions among cortisol, testosterone, and estradiol are correlated with heightened risk of anxiety and depression, with notable sex‐specific differences (Hazell et al. 2023, Chronister et al. 2021).

In females, elevated levels of testosterone and dehydroepiandrosterone have been identified as significant risk factors for depression. These hormonal changes may influence brain structure and function through various mechanisms. For instance, higher testosterone levels have been correlated with increased right hippocampal volume in adolescent girls, and such volumetric changes may be linked to depressive symptomatology (Ellis et al. 2019). Moreover, pubertal hormone fluctuations may enhance amygdala reactivity to negative emotional stimuli and contribute to functional abnormalities in the anterior cingulate cortex (ACC) during emotional regulation processes (Westlund Schreiner et al. 2017, Somerville et al. 2010).

Advancements in MRI technology have enabled the investigation of the neurobiological underpinnings of adolescent depression across structural, functional, and metabolic domains. One study reported increased myelin content in white matter tracts, including the uncinate fasciculus and corpus callosum, among adolescent females with depression in regions implicated in emotional and cognitive processing (Ho et al. 2021). Xu et al. (2024) identified enhanced coupling between structural and functional connectivity across 22 brain subregions in individuals with depression, primarily involving the precuneus, insula, inferior parietal lobule, and pulvinar nucleus, indicating altered coordination within brain networks. In addition, adolescents exhibiting suicidal behaviors demonstrated decoupling of structural and functional connectivity in the parahippocampal gyrus, while those who had experienced major life events presented increased coupling in the frontolimbic system, particularly between the orbitofrontal cortex and amygdala (Jiang et al. 2020). Given the role of the frontolimbic system in emotional regulation, these findings indicate that external stressors may influence depression risk by disrupting brain network integration.

Adolescent depression has been associated with reduced sensitivity to reward and heightened self‐referential processing. Specifically, diminished caudate nucleus responses to reward are linked to anhedonia, while hyperactivation of the medial prefrontal cortex contributes to excessive self‐focus and negative rumination, together forming a neural profile characterized by “low reward responsiveness and elevated self‐reflection” (Forbes et al. 2010). Furthermore, MRI has demonstrated superior predictive accuracy compared to individual clinical indicators in identifying the onset of depression, particularly among high‐risk groups like those with a family history of the disorder. Resting‐state functional connectivity has indicated notable predictive value in this context (Gracia‐Tabuenca et al. 2023).

The development of depression in adolescent females is influenced by a complex interplay of hormonal changes, social adaptation, and neurodevelopmental processes. MRI has emerged as a valuable modality for explaining the underlying pathological mechanisms, allowing for integrated analyses across structural, functional, and metabolic dimensions. Future research should prioritize longitudinal studies to examine the causal relationships between brain developmental trajectories and depression risk during adolescence, with the goal of facilitating early identification and intervention.

### 3.2 Perinatal Depression

Perinatal depression refers to depressive episodes occurring during pregnancy or within the first year postpartum. Clinical manifestations include low mood, anxiety, irritability, sleep disturbances, and diminished interest in the infant. In severe cases, individuals may experience thoughts of self‐harm or harm toward the infant (Van Niel et al. 2020). The pronounced hormonal fluctuations associated with pregnancy and childbirth, coupled with psychosocial stressors like role transitions and changes in social support, contribute to an increased risk of depression during the perinatal period (Bjelica et al. 2018).

From a biological standpoint, studies have reported that individuals with perinatal depression exhibit lower cortisol levels at specific time points, like early morning, compared to healthy controls, a pattern consistent with findings in non‐perinatal depression (Szpunar and Parry 2018, de Rezende et al. 2016, O’Connor et al. 2014). During pregnancy, estradiol and progesterone levels increase sharply, followed by a rapid decline postpartum. These hormonal fluctuations are closely associated with depressive and anxiety symptoms (Dukic et al. 2024). For instance, elevated progesterone levels have been correlated with greater severity of postpartum depressive symptoms, while higher estradiol concentrations appear to be linked to reduced anxiety symptoms. Additionally, within 24 to 48 h postpartum, individuals with serum testosterone levels exceeding 42.71 ng/mL had an increased risk of developing postpartum depression, although no significant differences in estradiol or progesterone levels were observed (Aswathi et al. 2015). These findings indicate that testosterone may contribute to postpartum depression by influencing emotion‐related brain regions, including the amygdala, hippocampus, and prefrontal cortex. Early indication of elevated testosterone levels may assist in identifying individuals at increased risk.

MRI has provided valuable insights into the neural mechanisms underlying perinatal depression. Prior research has demonstrated reduced functional connectivity in brain regions associated with emotional regulation, including the ACC, amygdala, hippocampus, and dorsolateral prefrontal cortex, among individuals with postpartum depression (Deligiannidis et al. 2013). With resting‐state fMRI, Chase identified decreased connectivity between the posterior cingulate cortex and amygdala in postpartum depression, indicating impaired integration within emotional regulation networks (Chase et al. 2014). Furthermore, when exposed to negative emotional stimuli, individuals with postpartum depression exhibited reduced activity in the amygdala and dorsomedial prefrontal cortex, reflecting a neural pattern characterized by increased sensitivity to negative emotions and diminished top‐down regulatory control (Moses‐Kolko et al. 2010).

Combined imaging modalities, including sMRI, fMRI, and MRS, have identified additional neural abnormalities. When compared to healthy postpartum individuals, those with postpartum depression demonstrated reduced gray matter volume in the prefrontal cortex, insula, hippocampus, and thalamus. During emotional processing tasks, there was reduced activation in the right insula, middle frontal gyrus, and inferior frontal gyrus. At rest, functional connectivity between the ACC and both the amygdala and dorsolateral prefrontal cortex was reduced (Duan et al. 2017). DTI studies further indicated decreased fractional anisotropy in the left anterior limb of the internal capsule, indicating compromised microstructural integrity of white matter and providing structural evidence of disruptions in emotion regulation pathways (Silver et al. 2018).

The pathogenesis of perinatal depression is shaped by the combined effects of hormonal fluctuations and changes in brain network regulation. Multimodal MRI findings indicate that perinatal depression is characterized by reduced functional connectivity in key emotional circuits, structural changes in gray matter, and abnormalities in white matter integrity.

### 3.3 Depression in Perimenopausal Women

Perimenopause is a natural transitional phase marking the shift from reproductive to post‐reproductive life, characterized by a progressive decline in ovarian function and corresponding reductions in estrogen and progesterone levels. Common symptoms during this period include hot flashes, night sweats, sleep disturbances, and mood fluctuations (Santoro et al. 2015). Along with hormonal changes, the presence of comorbid somatic symptoms like vasomotor disturbances, insomnia, and cardiovascular conditions may further elevate the risk of depression among perimenopausal women (Soares 2014). This stage has been identified as a period of heightened vulnerability to depressive disorders (Studd 2015).

As the most physiologically active form of estrogen, estradiol exerts its effects through dual mechanisms: on the one hand, its lipophilic nature enables it to cross the blood‐brain barrier and bind to estrogen response elements within the promoter regions of target genes via nuclear receptors, thereby regulating gene transcription; on the other hand, estradiol can also rapidly modulate neuronal activity through membrane receptors (Nathan et al. 2026, Liu et al. 2025). This dual mechanism allows estradiol to exert widespread influence on neurotransmitter systems throughout the brain, and may explain why increased sensitivity to estradiol fluctuations is observed during perimenopause (Parry 2021). Higher estradiol variability is positively associated with greater severity of depressive symptoms, a relationship thought to be mediated through changes in dopaminergic and serotonergic neurotransmitter system regulation (Joffe et al. 2020). Emerging evidence indicates a role for gut microbiota in modulating estrogen levels; for example, certain strains like *Klebsiella* spp. have been implicated in estradiol degradation, potentially contributing to decreased serum estrogen concentrations and an increased risk of depression onset (Li et al. 2023). Some studies have also shown that estrogen can enhance serotonin transmission. By binding to estrogen receptors, it upregulates the transcription of tryptophan hydroxylase‑2, the rate‑limiting enzyme in serotonin synthesis (Walsh et al. 2025). Estrogen can also affect serotonin reuptake by interacting with the serotonin transporter (Nathan et al. 2026). During perimenopause, the decline in estrogen reduces serotonin levels, leading to impaired serotonin transmission in key brain regions for emotional regulation, such as the prefrontal cortex, anterior cingulate cortex, and amygdala, thereby promoting the occurrence and development of depressive symptoms (Nathan et al. 2026, Wang et al. 2024). Furthermore, both the amplitude of estradiol fluctuations and the testosterone‐to‐estradiol ratio have been identified as potential predictive markers of depression risk during the perimenopausal period (Sander et al. 2021, Gordon et al. 2021).

MRI has identified structural, functional, and metabolic changes in perimenopausal women with depression. The sMRI studies have indicated reductions in both gray and white matter volumes, with the most prominent changes observed in the hippocampal region. The fMRI investigations have demonstrated increased spontaneous neural activity in regions like the lingual gyrus and precentral gyrus, along with decreased activity in the inferior temporal gyrus and putamen (Lu et al. 2023). In task‐based paradigms, enhanced activation has been noted in areas including the temporoparietal junction. During the processing of negative emotional stimuli, increased activation has been observed in brain regions involved in cognitive‐affective integration, including the prefrontal cortex, hippocampus, posterior cingulate cortex, and temporoparietal junction (Berent‐Spillson et al. 2017).

Metabolic assessments using MRS have indicated reduced glutamate concentrations in the prefrontal cortex and a gradual decline in γ‐aminobutyric acid levels in the ACC, findings that are closely associated with symptoms of depression and anxiety (Tran et al. 2023). Allopregnenolone, a metabolite of progesterone, is a potent positive allosteric modulator of the γ‑aminobutyric acid (GABA) receptor (Stefaniak et al. 2023). Reduced GABA levels during perimenopause may be associated with fluctuations in allopregnenolone (Crockett et al. 2025). Decreased levels of allopregnenolone can lead to hyperactivation of the amygdala, thereby affecting emotional regulation (Stefaniak et al. 2023, Wang et al. 2019). DTI studies have reported decreased fractional anisotropy in the insula and midbrain white matter, indicating compromised white matter integrity in emotion regulation pathways (Wang et al. 2014). Additionally, research involving perimenopausal individuals with burning mouth syndrome has presented abnormalities in both the structure and connectivity patterns of the hippocampus and prefrontal cortex, indicating that chronic pain and affective disorders may share overlapping neural mechanisms (Khan et al. 2014).

The pathogenesis of depression in perimenopausal women involves a multifactorial interplay of hormonal fluctuations, dysregulation of neurotransmitter systems, and structural and functional brain abnormalities. MRI studies have identified widespread changes in key regions implicated in emotional regulation, particularly the hippocampus, prefrontal cortex, and ACC. These changes are reflected in structural imaging findings, disruptions in white matter tract integrity, and changes in metabolic parameters, collectively providing neurobiological markers of perimenopausal depression.

### 3.4 Late‐Life Depression in Women

Late‐life depression (LLD) commonly affects individuals aged 60 years and older, with a higher prevalence observed among women. It is frequently associated with postmenopausal estrogen deficiency, increased burden of chronic medical conditions, and diminished social support (Luppa et al. 2012, Kraaij et al. 2002). Studies have found that chronic stress is a significant pathogenic factor of depression in elderly women. Chronic stress can induce hyperactivity of the hypothalamic‐pituitary‐adrenal axis, leading to sustained elevation of cortisol. In elderly women, serum cortisol levels are positively correlated with the severity of depression, whereas no such correlation exists in elderly men (Winczyk et al. 2022). In comparison to men, older women exhibit a greater prevalence of comorbid psychiatric disorders (Laborde‐Lahoz et al. 2015). Elevated levels of testosterone and cortisol may exacerbate depressive symptoms, while chronic stress can activate the hypothalamic–pituitary–adrenal axis and impair its negative feedback regulation (Winczyk et al. 2022). Genetic polymorphisms in *CYP19A1*, which encodes the aromatase enzyme, may disrupt sex hormone homeostasis by altering enzymatic activity, thereby increasing susceptibility to depression (Ancelin et al. 2020). Additionally, lower serum concentrations of testosterone and estrone have been associated with depressive symptoms in older women (Islam et al. 2023).

The sMRI and DTI studies have indicated brain abnormalities in older women with depression. These include global cerebral atrophy and reduced gray matter volume in regions like the right straight gyrus, left middle temporal gyrus, left orbital inferior frontal gyrus, and hippocampus. Abnormalities in white matter and decreased functional connectivity have been reported (Patel et al. 2015, Zhao et al. 2024). DTI findings indicate reduced fractional anisotropy in major white matter tracts, including the corpus callosum, cingulum bundle, and anterior limb of the internal capsule, indicating compromised microstructural integrity. An increased burden of white matter hyperintensities, particularly within frontolimbic circuits, has been proposed as a potential imaging biomarker of vascular depression (Patel et al. 2015, Emsell et al. 2017). These structural changes reflect neurodegenerative features of LLD and may contribute to its overlap with neurodegenerative disorders such as Alzheimerne disease.

Resting‐state fMRI studies in older women with depression have demonstrated decreased functional connectivity within the default mode network, specifically between the ACC, hippocampus, and orbitofrontal cortex regions integral to internal cognition and emotional regulation. Severity of depressive symptoms has been correlated with reduced connectivity between the right ACC and the superior frontal gyrus. Multimodal imaging studies combining sMRI, fMRI, and DTI have indicated that structural and functional abnormalities frequently co‐occur in the same brain regions, particularly in the ACC and hippocampus. This convergence highlights structural–functional decoupling as a central neurobiological mechanism underlying late‐life depression in women (Patel et al. 2015, Tudorascu et al. 2014, Tadayonnejad et al. 2014).

Depression in older women is shaped by a combination of sex hormone alterations, neurodegenerative processes, vascular pathology, and psychosocial stressors. MRI has indicated widespread structural and functional brain abnormalities, including gray and white matter atrophy, compromised microstructural integrity, reduced connectivity within networks involved in emotional regulation, and structural–functional decoupling.

A 2025 study further underscores these dynamic changes, indicating that structure‑function coupling is an important factor in predicting late‑life depression and cognitive decline. For instance, multimodal imaging studies have shown that elderly women in a subsyndromal depressive state exhibit significantly weakened structure‑function connectivity coupling compared with healthy controls, and this decoupling is negatively correlated with the severity of depressive symptoms (Suo et al. 2025). Another study demonstrated that in individuals with late‑life depression, key brain networks—including the visual network, ventral attention network, and default mode network—show increased intranetwork connectivity, with enhanced static functional connectivity being particularly prominent in the ventral attention network (Li et al. 2025). Furthermore, in late‐life depression, estrogen exerts neuroprotective effects by regulating the synthesis and metabolism of serotonin. The significantly decreased estrogen levels in patients with late‐life depression result in the loss of such protective effects, which may exacerbate neuroinflammatory pathways and thereby increase the risk of developing late‐life depression (Ryan et al. 2011).

Future research should focus on elucidating sex‐specific mechanisms and advancing the use of MRI in high‐risk population screening, treatment response prediction, and the development of targeted intervention strategies.

To provide a comprehensive overview, Table 1 presents a summary of key neuroimaging findings and hormone‐related factors associated with depression across different stages of the female lifespan.

**TABLE 1:** Summary of neuroimaging findings, methods, and hormonal correlates of depression across the female lifespan.

Life stage and references	Methods and techniques	Key findings	Hormonal changes and effects
Adolescence (Angold et al., 1999, Hazell et al., 2023, Chronister et al., 2021, Ellis et al., 2019, Westlund Schreiner et al., 2017, Somerville et al., 2010, Ho et al., 2021, Xu et al., 2024, Jiang et al., 2020, Forbes et al., 2010, Gracia‐Tabuenca et al., 2023)	qMRI + DTI; fMRI; resting‐state fMRI	‐ Increased myelin in cingulum and corpus callosum ‐ Reduced reward sensitivity (caudate nucleus hypoactivity) ‐ Increased medial prefrontal cortex activity (self‐focus) ‐ Altered SC‐FC coupling in multiple brain regions, especially limbic circuits	‐ Testosterone >24.7 ng/dl increases depression risk ‐ Testosterone correlates with right hippocampal volume ‐ Hormonal fluctuations intensify amygdala and anterior cingulate cortex, responses ‐ Complex interactions among cortisol, testosterone, and estradiol heighten anxiety and depression risk
Peripartum (Szpunar and Parry, 2018, De Rezende et al., 2016, O'connor et al., 2014, Dukic et al., 2024, Aswathi et al., 2015, Deligiannidis et al., 2013, Chase et al., 2014, Moses‐Kolko et al., 2010, Duan et al., 2017, Silver et al., 2018)	fMRI; sMRI + fMRI + MRS; DTI	‐ Reduced functional connectivity in ACC, amygdala, hippocampus ‐ Gray matter volume loss in prefrontal cortex, amygdala, hippocampus ‐ Reduced fractional anisotropy in left anterior limb of internal capsule	‐ High progesterone linked to more severe depression ‐ Postpartum testosterone >42.71 ng/ml increases depression risk ‐ Lower morning cortisol levels observed ‐ Placental CRH creates positive feedback loop elevating cortisol during pregnancy with sharp decline postpartum
Perimenopause (Nathan et al., 2026, Liu et al., 2025, Parry, 2021, Joffe et al., 2020, Li et al., 2023, Walsh et al., 2025, Wang et al., 2024, Sander et al., 2021, Gordon et al., 2021, Lu et al., 2023, Berent‐Spillson et al., 2017, Tran et al., 2023, Stefaniak et al., 2023, Crockett et al., 2025, Wang et al., 2019, Wang et al., 2014, Khan et al., 2014)	dMRI; fMRI; MRS; sMRI + DTI + rs‐fMRI	‐ Decreased GMV and WMV, especially hippocampus ‐ Microstructural white matter alterations (insula, midbrain) ‐ Increased spontaneous activity in frontal and temporal regions ‐ Decreased Glu and GABA levels in ACC ‐ Decreased activity in inferior temporal gyrus and putamen ‐ Enhanced activation in temporoparietal junction during negative emotional processing ‐ Decreased glutamate concentrations in prefrontal cortex	‐ Large fluctuations in estradiol increase depression severity ‐ High testosterone‐to‐estradiol ratio worsens symptoms ‐ Gut microbiota (Klebsiella) degrade estradiol ‐ Reduced allopregnenolone (progesterone metabolite) leads to amygdala hyperactivation ‐ Estrogen decline reduces serotonin transmission in prefrontal cortex, ACC, and amygdala
Older Age (Luppa et al., 2012, Kraaij et al., 2002, Winczyk et al., 2022, Laborde‐Lahoz et al., 2015, Ancelin et al., 2020, Islam R et al., 2023, Patel et al., 2015, Zhao et al., 2024, Emsell et al., 2017, Tudorascu et al., 2014, Tadayonnejad et al., 2014, Suo et al., 2025, Li et al., 2025, Ryan et al., 2011)	fMRI; T1‐weighted MRI; WMH quantification; sMRI + DTI + fMRI	‐ Reduced connectivity in right nucleus accumbens to medial orbitofrontal cortex, ACC to superior frontal gyrus ‐ Gray matter atrophy in hippocampus, ACC, frontal regions ‐ White matter integrity loss and increased WMH load ‐ Default mode network connectivity reduction	‐ Increased testosterone and cortisol linked to depressive symptoms ‐ Low testosterone and estrone increase depression risk ‐ Chronic stress induces HPA axis hyperactivity with sustained cortisol elevation ‐ Loss of estrogen neuroprotective effects exacerbates neuroinflammatory pathways

### 3.5 Integrative Biopsychosocial Model of Women's Depression Across the Lifespan

Adolescent depression arises from the interplay of pubertal hormones, early adversity, and current psychosocial stress. Li et al. found that depressed adolescents who experienced early life stress—such as abuse, peer bullying, and family adversity—exhibit hypermethylation of the neuron‐specific glucocorticoid receptor gene in the hippocampus. Such hypermethylation of the glucocorticoid receptor gene may disrupt the function of the hypothalamic‐pituitary‐adrenal (HPA) axis, rendering these adolescents more vulnerable to depression (Li et al. 2020).

Perinatal depression is not caused by a single factor, but rather results from the dynamic interaction between psychosocial factors and the neuroendocrine system. During pregnancy, the placenta synthesizes corticotropin‐releasing hormone. Glucocorticoids inhibit the gene expression of hypothalamic corticotropin‐releasing hormone, but in the placenta, glucocorticoids promote its production, thereby forming a positive feedback loop. This leads to elevated cortisol levels during pregnancy and a sharp decline postpartum. Notably, pregnant individuals with a history of childhood trauma exhibit lower average cortisol levels and blunted stress responses compared with women without such trauma (Hantsoo et al. 2023). These dysregulations of the hypothalamic‐pituitary‐adrenal axis may increase vulnerability to depression. Neuroimaging further reveals that patients with perinatal depression show hyperactivation of the default mode network and abnormal functional connectivity between the anterior cingulate cortex and the amygdala (Horáková et al. 2022).

Perimenopausal women are also in a vulnerable period affected by the interaction of physiological, psychological, and social factors. The combined effects of chronic life stress (such as unemployment, lack of social support, and major life events) and drastic hormonal fluctuations during perimenopause reduce the function of γ‑aminobutyric acid receptors, leading to hyperactivation of the hypothalamic‑pituitary‑adrenal axis (Gordon et al. 2015). In addition, the function of the serotonin system mediated by estrogen receptors decreases (Crockett et al. 2025). The synergistic action of multiple pathways ultimately increases the susceptibility to depression.

Similarly for older women: Liu et al. (2025) found that social isolation significantly increased the risk of depression in elderly women. Regarding hormonal mechanisms, declining estrogen levels make the hypothalamic‐pituitary‐adrenal axis more sensitive to stress in older women (Winczyk et al. 2022). Neuroimaging studies have revealed that elderly women with subclinical depression exhibit increased activity in the left dorsolateral prefrontal cortex and decreased functional activity in the right precuneus (Jiang et al. 2025). The interaction of these factors makes older women more susceptible to depression than men.

## 4 Conclusion and Future Perspectives

This review synthesizes the application of MRI in the study of depression across various stages of the female lifespan, highlighting structural, functional, and metabolic brain abnormalities. These neuroimaging findings contribute to a deeper understanding of the underlying pathophysiological mechanisms and support efforts toward the early identification of depression in women. Despite these advances, current research is constrained by limitations like small sample sizes, cross‐sectional study designs, and insufficient integration of hormonal, psychological, and genetic variables.

Most existing literature has focused on depression vulnerability linked to natural hormonal fluctuations. However, neuroimaging studies on depression in women with endocrine disorders—including polycystic ovary syndrome and ovarian insufficiency—remain limited. Similarly, the complex neuroimaging mechanisms underlying exogenous hormone replacement therapy have yet to be fully elucidated. Moreover, the lack of racial, cultural, and socioeconomic diversity in neuroimaging cohorts constrains the generalizability of current findings.

Future studies should prioritize large‐scale, longitudinal, and multicenter studies that incorporate MRI along with hormonal and genomic data to further develop the biopsychosocial model of depression. MRI‐derived biomarkers offer significant potential for high‐risk population screening and personalized treatment, thereby improving the precision of diagnostic and therapeutic strategies. Advancing scientific knowledge and facilitating clinical translation will require sustained interdisciplinary collaboration.

## Author Contributions

**Rong Zi**: conceptualization, formal analysis. **Chongyi Zhao**: conceptualization, formal analysis, funding acquisition, writing – review and editing. **Zhirun Chen**: formal analysis, writing – original draft, investigation. **Ying Liu**: investigation, formal analysis, writing – original draft.

## Funding

This research was supported by Central Government Guidance Fund for Local Science and Technology Development (202407AB110013).

## Ethics Statement

Not applicable.

## Conflicts of Interest

The authors declare no conflicts of interest.

## Data Availability

The datasets used or analyzed during the current study are available from the corresponding author upon reasonable request.
